# Sensitivity and Specificity of a Novel Colony Characteristic for Determination of Methicillin-Resistant Staphylococcus aureus

**DOI:** 10.7759/cureus.26040

**Published:** 2022-06-17

**Authors:** Apeksha N Agarwal, Steven D Dallas, Daniel D Mais

**Affiliations:** 1 Pathology, University of Texas Health Science Center at San Antonio, San Antonio, USA; 2 Microbiology, University of Texas Health Science Center at San Antonio, San Antonio, USA; 3 Pathology and Laboratory Medicine, University of Louisville School of Medicine, Louisville, USA

**Keywords:** mrsa, target shaped, blood agar, colony morphology, methicillin resistant staphylococcus aureus, staphylococcus aureus

## Abstract

Purpose: To assess colony morphology of *Staphylococcus aureus* isolates for target shape (T1) and its utility in the identification of methicillin-resistant *S. aureus* (MRSA).

Methods: *Staphylococc*u*s* species isolated from blood cultures were studied for colony morphology characteristics. A polymerase chain reaction (PCR) test was performed on positive blood culture bottles for the detection of *S. aureus* and methicillin resistance. Colony morphology was read at 24 and 48 hours and defined as follows: target shaped (T1) - an elevated colony center encircled by a pale zone, which is surrounded by a single ring of peripheral enhancement giving a ‘target’ appearance; dome-shaped (T2) with an elevated center lacking the ‘target’ appearance.

Results: At 48 hours, 73.7% of MRSA and 59.5% of coagulase-negative staphylococci (CoNS) showed T1 morphology. T1 morphology has a sensitivity of 73.68% and specificity of 93.55% amongst *S. aureus* for identification of methicillin resistance and a high positive predictive value (95.45%) at 48 hours.

Conclusion: T1 morphology has a modest sensitivity with specificity and positive predictive value amongst *S. aureus* for identification of methicillin resistance at 48 hours. It can be potentially used for the identification of MRSA, especially in resource-limited settings and wherein a molecular test is not repeated if PCR testing has already identified methicillin-sensitive *S. aureus (*MSSA) on a recent specimen on the same patient.

## Introduction

*Staphylococcus aureus* is a gram-positive coccus that can colonize nares and is an opportunistic pathogen leading to diseases including pneumonia, endocarditis, and bacteremia [1]. The colonies show characteristic yellow, round, large (1-3 mm), convex, and opaque colonies with beta hemolysis. Based on oxacillin resistance they are characterized as methicillin-resistant *S. aureus* (MRSA) or methicillin-sensitive *S. aureus* (MSSA). MRSA can produce non-hemolytic and white colonies, which resemble coagulase-negative staphylococci (CoNS). MRSA is a multi-drug resistant pathogen with resistance to penicillins and cephalosporins [2]. In this study, we reviewed *S. aureus* isolates for a novel colony morphology characteristic, which can aid in the identification of MRSA.

The article was presented as a poster at World Microbe Forum, 20-24 June 2021.

## Materials and methods

This is a prospective study in which *Staphylococcus* species isolated from blood cultures were included. No identifiable information was collected during the study period and consent was waived by the institutional review board of the University of Texas Health, San Antonio (UTHSA), Texas, United States.

**Figure 1:**
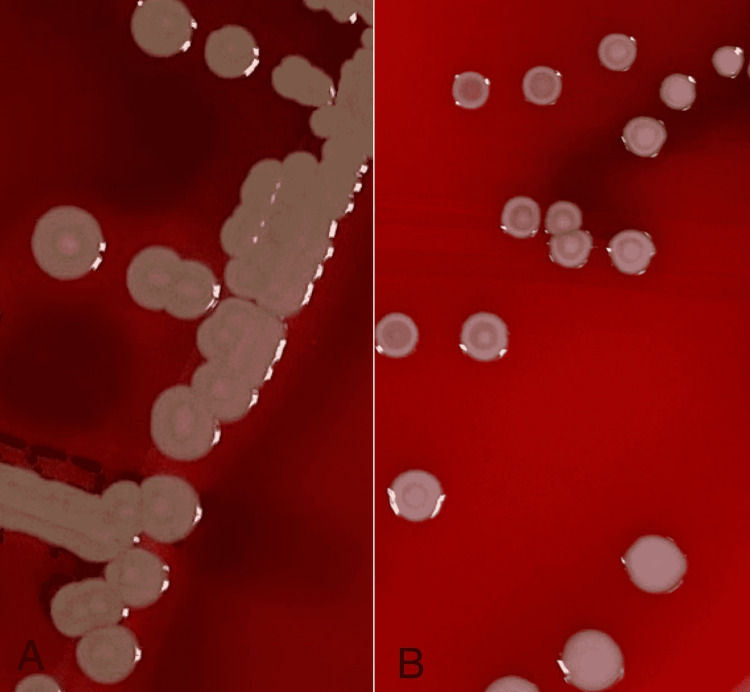
Target-shaped (T1) colony morphology: (A) in a MRSA isolate; (B) in a CoNS isolate MRSA: methicillin-resistant *Staphylococcus aureus*; CoNS: coagulase-negative staphylococci

**Figure 2:**
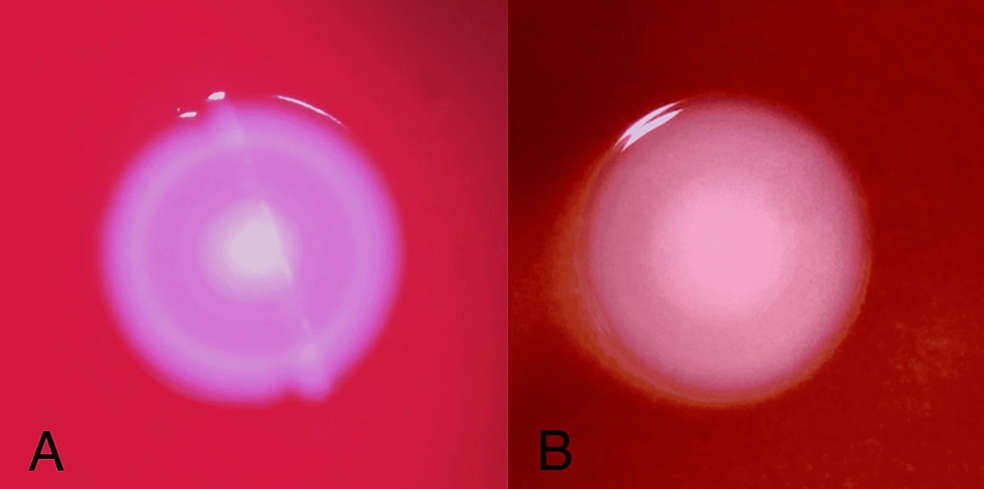
(A) Colony of CoNS observed under the dissecting microscope showing target shape (T1); (B) Colony of MRSA observed under the dissecting microscope showing target shape (T1) MRSA: methicillin-resistant *Staphylococcus aureus*; CoNS: coagulase-negative staphylococci

**Figure 3:**
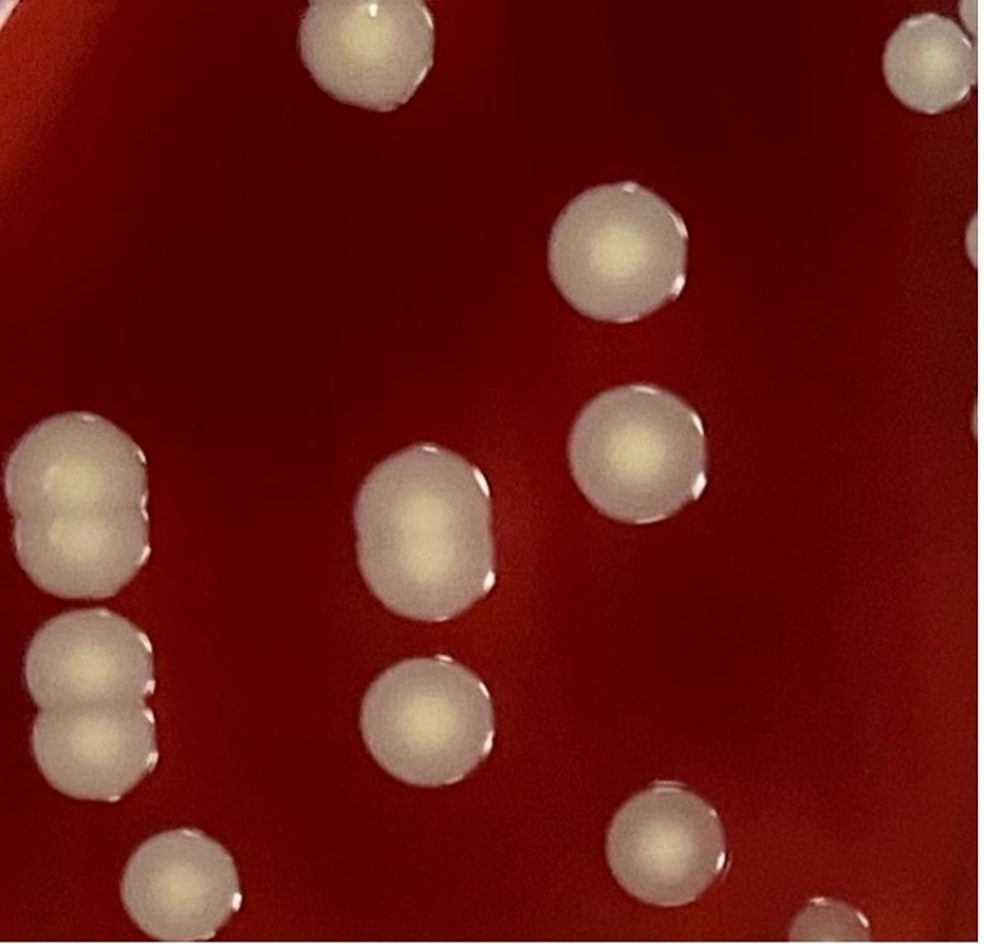
Dome-shaped (T2) colony morphology in Staphylococcus aureus lacking the target shape

Inclusion criteria were well-isolated colonies of *S. aureus *on blood agar plates and exclusion criteria were small colonies and confluent colonies on blood agar plates. Blood agar plates were incubated for 24 and 48 hours at 35 °C with 5% CO2. The plates were read with the naked eye. If needed, a magnifying glass was used for assistance in visualizing the colonies. Colony shapes were classified as target-shaped (T1) or dome-shaped (T2). T1 is an elevated colony center encircled by a pale zone, which is surrounded by a single ring of peripheral enhancement giving a ‘target’ appearance (Figure 1). Magnified images of T1 colonies in CoNS and MRSA under dissecting microscope are depicted in Figure 2A and Figure 2B, respectively. T2 colony is with an elevated center and lacks the ‘target’ appearance (Figure 3).

A commercially available PCR test (Cepheid® Xpert MRSA/SA Blood Culture test; Cepheid, Sunnyvale, California, United States) was performed on all first positive *S. aureus* blood culture bottles for the confirmation of *S. aureus* and detection of methicillin resistance.

## Results

A total of 239 cases were screened for the study. Forty isolates were excluded from the study as the colonies were tiny or confluent, which prohibited the evaluation of colony morphology. After the exclusion of cases, a total of 199 *Staphylococcus* isolates were included in the study. Of the *Staphylococcus* isolates, 111 isolates were CoNS species. Of the remaining 88, 57 were MRSA and 31 were MSSA.

The colony morphologies recorded at 24 hours and 48 hours for target shape were as follows: At 24 hours, 19.3% of MRSA, 0.06% of MSSA, and 5.4% of CoNS had T1 colony morphology. At 48 hours, 73.7% of MRSA and 59.5% of CoNS showed T1 morphology as depicted in Table 1. T1 colony morphology has a sensitivity of 73.68% and specificity of 93.55% amongst *S. aureus* to detect MRSA strains at 48 hours with a positive predictive value of 95.45% (95%CI 84.49-98.78%).

**Table 1:** Number of isolates showing target-shaped (T1) and dome-shaped (T2) colony morphology of Staphylococcus species at 48 hours MRSA: methicillin-resistant *Staphylococcus aureus*; MSSA: methicillin-sensitive *Staphylococcus aureus*; CoNS: coagulase-negative staphylococci

Organism (n)	Target-shaped (T1)	Dome-shaped (T2)	Neither T1 or T2
MRSA (57)	42	13	2
MSSA (31)	2	27	2
CoNS (111)	66	0	45

## Discussion

The rapid identification of MRSA can be performed by fluorescence in situ hybridization (ISH), microarray, multiplex PCR, real-time PCR, and mass spectrometry. Identification can also be performed from positive blood culture bottles [3-12]. These tests may be cost-prohibitive in some settings. Traditional, less expensive but slower identification techniques include culture and subsequent confirmation of MRSA by disk diffusion and broth microdilution susceptibility testing.

A commonly used phenotypic method is screening isolated colonies by disk diffusion. Oxacillin and cefoxitin disks are most often used for screening. However, cefoxitin is recommended for screening as it can induce mecA and mecC better than oxacillin [13,14]. Chromogenic agar plates may be used to detect MRSA. The sensitivity of tests varied amongst different studies. In a study by Cesur et al., oxacillin resistance screen agar base (ORSAB) and CHROMagar™ MRSA media (Kanto Chemical Co. Inc., Tokyo, Japan) were compared to the oxacillin disk diffusion test reference method [15]. ORSAB had a sensitivity, specificity, and positive predictive value of 97.7%, 40%, and 36.5%, respectively. CHROMagar MRSA had a sensitivity, specificity, and positive predictive value of 95.5%, 37.6%, and 35.7% respectively. In a study by Onze-Lieve-Vrouwziekenhuis et al., MRSA II agar had a sensitivity and specificity of 98.9% and 89.4%, respectively, at 48 hours whereas MRSA ID had a sensitivity and specificity of 98.2% and 84.7%, respectively, at 48 hours respectively [16].

Other alternative methods have been studied for the identification of MRSA in histopathologic sections. These include immunohistochemistry (IHC) and ISH studies. IHC studies utilize the species-specific antigens of *S. aureus* and Penicillin-Binding Protein 2 (PBP2′) for identification [6]. MRSA can also be identified with ISH by demonstration of both the species-specific gene and mecA. However, these studies were predominantly performed on nonarchival sections such as unfixed frozen sections or paraformaldehyde-FFSs [17].

A literature search for T1 colony morphology on blood agar plates for the identification of MRSA was conducted. However, no similar studies were identified.

Limitations

**Figure 4:**
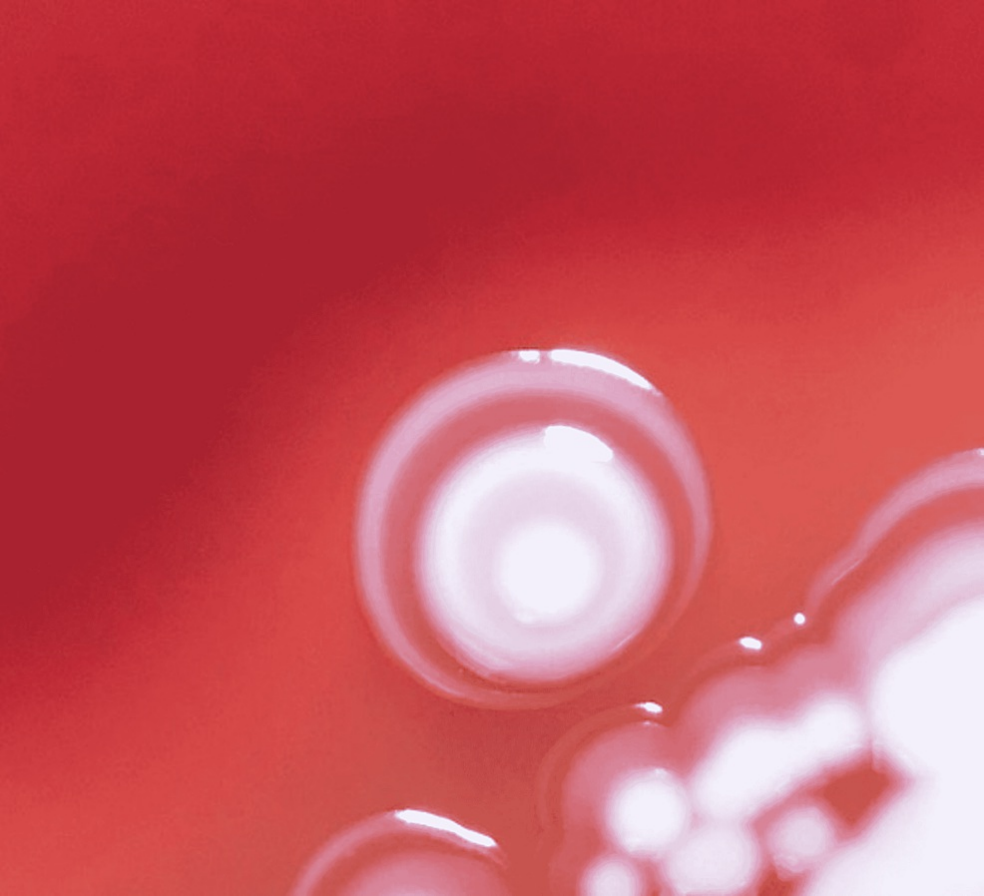
Colony of MSSA showing two zones of enhancement with an elevated colony center at 48 hours (deviating from defined target shape) MSSA: methicillin-sensitive *Staphylococcus aureus*

First, care should be taken to read the morphology in well-isolated colonies. Confluent colonies may be falsely read as target shaped (T1) colony. Second, colony morphology at 48 hours should be considered for final results as the sensitivity increased from 19.3% to 73.68% for detection of MRSA at 48 hours; MSSA can, in rare cases, show a T1 morphology at 24 hrs. For example, on further incubation to 48 hours, a double zone of enhancement can be observed (deviating from the defined T1 shape colony) as in Figure 4.

## Conclusions

T1 morphology has a modest sensitivity with a specificity and positive predictive value amongst *S. aureus* for identification of methicillin resistance at 48 hours. T1 colony morphology at 48 hours can be potentially used for the identification of MRSA, especially in resource-limited settings as it does not require additional reagents, antibiotic disks, or media for detection.

T1 morphology can also be utilized in a setting where a molecular test is not repeated if PCR testing has already identified MSSA on a recent specimen on the same patient. Isolates can be further incubated for 24 hours to detect T1 colony morphology, which is suggestive of MRSA. Thereby, only cases showing T1 colonies at end of 48 hours can be further tested for confirmation by additional methods such as PCR or latex agglutination, improving patient care. The T1 colony morphology adds to the colony morphologic characteristics of MRSA.
